# Identification of suitable internal control genes for expression studies in *Coffea arabica *under different experimental conditions

**DOI:** 10.1186/1471-2199-10-1

**Published:** 2009-01-06

**Authors:** Carla F Barsalobres-Cavallari, Fábio E Severino, Mirian P Maluf, Ivan G Maia

**Affiliations:** 1 Laboratório de Biotecnologia e Genética Molecular, Departamento de Genética, Instituto de Biociências, UNESP, Distrito de Rubião Júnior s/n, 18618-000, Botucatu, São Paulo, Brazil; 2 Embrapa/IAC, Centro de Café Alcides Carvalho, Campinas, São Paulo, Brazil

## Abstract

**Background:**

Quantitative data from gene expression experiments are often normalized by transcription levels of reference or housekeeping genes. An inherent assumption for their use is that the expression of these genes is highly uniform in living organisms during various phases of development, in different cell types and under diverse environmental conditions. To date, the validation of reference genes in plants has received very little attention and suitable reference genes have not been defined for a great number of crop species including *Coffea arabica*. The aim of the research reported herein was to compare the relative expression of a set of potential reference genes across different types of tissue/organ samples of coffee. We also validated the expression profiles of the selected reference genes at various stages of development and under a specific biotic stress.

**Results:**

The expression levels of five frequently used housekeeping genes (reference genes), namely *alcohol dehydrogenase *(*adh*), *14-3-3*, *polyubiquitin *(*poly*), *β-actin *(*actin*) and *glyceraldehyde-3-phosphate dehydrogenase *(*gapdh*) was assessed by quantitative real-time RT-PCR over a set of five tissue/organ samples (root, stem, leaf, flower, and fruits) of *Coffea arabica *plants. In addition to these commonly used internal controls, three other genes encoding a cysteine proteinase (*cys*), a caffeine synthase (*ccs*) and the 60S ribosomal protein L7 (*rpl7*) were also tested. Their stability and suitability as reference genes were validated by geNorm, NormFinder and BestKeeper programs. The obtained results revealed significantly variable expression levels of all reference genes analyzed, with the exception of *gapdh*, which showed no significant changes in expression among the investigated experimental conditions.

**Conclusion:**

Our data suggests that the expression of housekeeping genes is not completely stable in coffee. Based on our results, *gapdh*, followed by *14-3-3 *and *rpl7 *were found to be homogeneously expressed and are therefore adequate for normalization purposes, showing equivalent transcript levels in different tissue/organ samples. *Gapdh *is therefore the recommended reference gene for measuring gene expression in *Coffea arabica*. Its use will enable more accurate and reliable normalization of tissue/organ-specific gene expression studies in this important cherry crop plant.

## Background

The study of biological regulations is very often correlated to quantification assays. In order to detect differential expression of a gene(s) in distinct biological samples, such as tissue types or under different experimental conditions, the invention of quantitative PCR (qPCR) has transformed the field of gene expression analysis in living organisms [1]. In comparison to classical reverse transcription-polymerase chain reaction (RT-PCR), the main advantages of qPCR are higher sensitivity, specificity and broad quantification range of up to seven orders of magnitude [2-6]. Regardless of being an extremely powerful technique, qPCR has its pitfalls, the most important one being the need of appropriate data normalization with a reference gene [6-14].

According to Andersen et al. [8], accurate data normalization is an absolute requirement for correct measurement of gene expression. Expression of the reference gene used to normalize qPCR analyses should be unaffected throughout many biological contexts; otherwise, it may lead to erroneous results [6-9,15-18]. Until recently, several such genes (*β-actin*, *rRNA*, *β-tubulin*, *alcohol dehydrogenase*, *glyceraldehyde-3-phosphate dehydrogenase*, *14-3-3 *and *polyubiquitin*) have been used as internal controls for gene expression analyses under the assumption of stable expression [19-26]. However, several reports have demonstrated that the expression levels of these so-called reference genes differ among different tissue/organ types [10,16,27-33]. Consequently, these genes are unsuitable as transcriptional inner controls, and their use to normalize qPCR data in different tissues may induce significant experimental errors that could result in inappropriate biological data interpretation [9,14,17,21-25,34-39].

Recognizing the importance of reference gene(s) in normalization of qPCR data, various housekeeping genes have been evaluated for stable expression under specific conditions in various organisms. In plants, only a few of them have been investigated in some detail in rice [15,40,41], poplar [36], potato [39], soybean [42,43] and *Arabidopsis thaliana *[30]. So far, suitable internal controls for gene expression studies have not been defined for *Coffea arabica*.

Coffee is an agricultural crop of significant economic importance. *Coffea arabica *L. (arabica type coffee) is typical of the highland growing regions and is responsible for almost 75% of world production [44]. In this study, we report the validation of housekeeping genes to identify the most suitable internal reference gene(s) for normalization of qPCR data obtained among five different tissues/organs (root, stem, leaf, flower, and fruits) of *C. arabica*. To further validate our results, we evaluated the expression levels of our best reference genes at different developmental stages of flowers and cherries and under a specific biotic stress. Following the current literature, five candidate reference genes, namely *alcohol dehydrogenase *(*adh*), *polyubiquitin *(*poly*), *14-3-3*, *β-actin *(*actin*) and *glyceraldehyde-3-phosphate dehydrogenase *(*gapdh*), were selected. In addition to these commonly used internal controls, three other genes coding for a cysteine proteinase (*cys*), a caffeine synthase (*ccs*) and the 60S ribosomal protein L7 (*rpl7*), respectively, were included in this analysis. These potential reference genes were ranked according to their expression profiles and stability.

## Results and discussion

The expression profile of eight candidate reference genes (*actin*, *adh*, *14-3-3*, *ccs*, *gapdh*, *poly*, *rpl7*, or *cys*) was firstly assessed by qPCR over a panel of five coffee tissue/organ samples (root, stem, leaf, flower, or fruit).

### Descriptive analysis of the reference candidate genes

Descriptive statistics of the derived crossing points (CP), based on BestKeeper program [45], were calculated to investigate the variation level of each candidate gene following Pfaffl et al. [46]. According to this analysis (see Table 1), the gene with lowest expression level was *actin*, for which CP values were obtained around cycles 31–34; while the highest was *gapdh*, whose CP values were obtained around cycles 21–23. The expression levels of *14-3-3*, *ccs*, *gapdh*, and *rpl7 *presented fluctuations of approximately ± 0.6 x-fold (0.52 x-fold < SD < 0.82 x-fold), whereas *poly *expression showed higher ranges of CP variation (SD = ± 1.39 cycles) as well as up- down-regulation (± 2.09 x-fold). The coefficient of variation (CV) of the assay was 3.57% (total essay variability), which is within the range (from 3.4% to 11.6%) of previously reported values for qPCR [46].

**Table 1:** Descriptive statistics and expression level analyses of the tested candidate reference genes based on their crossing point (CP) values

Factor	*actin*	*adh*	*14-3-3*	*ccs*	*gapdh*	*poly*	*rpl7*	*cys*
N	21	21	21	21	21	21	21	21
GM [CP]	32.72	29.7	29.63	26.67	22.86	28.46	30.76	26.67
AM [CP]	32.74	29.71	29.64	26.67	22.88	28.49	30.77	26.7
Min [CP]	31.4	28.64	28.94	25.92	21.74	26.79	30.13	25.24
Max [CP]	34.35	31.12	31.21	27.37	23.77	30.07	31.93	28.44
SD [± CP]	1.23	0.99	0.92	0.58	0.94	1.39	0.69	1.31
CV [%CP]	3.75	3.35	3.09	2.19	4.12	4.89	2.24	4.89
								
Min [x-fold]	0.41	0.43	0.66	0.53	0.54	0.18	0.68	0.41
Max [x-fold]	3.04	3.1	2.62	1.83	1.64	5.22	2.02	3.04
SD [± x-fold]	1.07	1.08	0.82	0.52	0.52	2.09	0.53	1.08

Abbreviations: N: number of samples; CP: crossing-point; GM [CP]: geometric CP mean; AM [CP]: arithmetic CP mean; Min [CP] and Max [CP]: CP threshold values; SD [± CP]: CP standard deviation; CV [%CP]: variance coefficient expressed as percentage of CP level; Min [x-fold] and Max [x-fold]: threshold expression levels expressed as absolute x-fold over- or under-regulation coefficient; SD [± x-fold]: standard deviation of absolute regulation coefficient.

Descriptive statistics for the expression analyses of each reference candidate gene in the five distinct coffee plant tissue/organ types was also obtained (see Additional File 1). According to this analysis, the gene that exhibit the minor gene expression variation among the analyzed tissues was *gapdh *[SD (± x-fold) = 0.04], while the gene with major variation was *ccs *[SD (± x-fold) = 0.59]. In addition, *ccs *also presented the highest expression variation in flowers and fruits tissue samples, and therefore it cannot be used as a reference gene. Numerous studies have shown that the expression of housekeeping genes can vary under given situations [28]. This may partly be explained by the fact that housekeeping genes are not only implicated in the basal cell metabolism but also participate in other cellular functions [47,48].

### A model-based approach for estimation of expression variation

**Figure 1:**
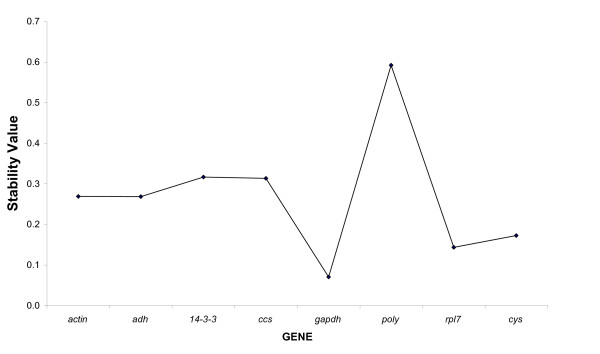
**Stability of the investigated candidate reference genes**. Stability values of the eight candidate reference genes according to the model-based approach. A lower value of average expression stability indicates more stable expression.

The model-based variance estimation approach, a Visual Basic application for Microsoft Excel (termed NormFinder) [8,49], was used to evaluate expression stability of reference candidate genes. This analysis allowed the ranking of candidate genes since the estimated variation directly indicates the introduced error associated with their use. According to this method, the gene with minimal estimated intra- and intertissue variation was *gapdh *(expression stability = 0.071) while the gene with the maximal variation was *poly *(expression stability = 0.592) (see Figure 1), thus corroborating the results obtained in descriptive analysis.

### Ranking the candidate reference genes

**Figure 2:**
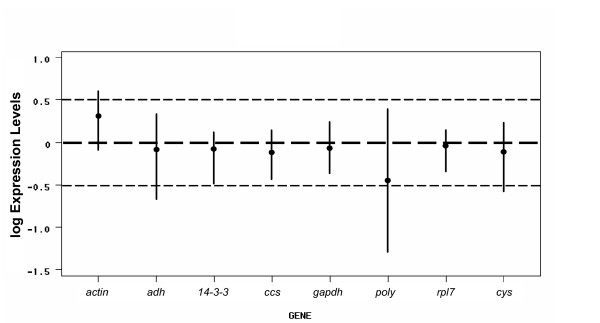
**Gene expression differences among the candidate reference genes**. The log-transformed gene expression levels are represented by black circles. The intertissue variation is indicated by vertical bars that give a confidence interval for the difference. The two thin dashed lines represent the maximal standard deviation of the reference candidate genes, with a log expression levels difference between 0.5 and -0.5.

The relationship between the stability value and the intra- and intertissue expression variations is present in Figure 2. This figure clearly demonstrates the distinct specificities of the investigated genes. According to this analysis, the best candidate gene should present the minimal combined inter- and intra-tissue expression variation. Consistent with the descriptive analysis (see Table 1 and Additional File 1) and the model-based variance estimation approach (see Figure 1), *gapdh *showed not only the highest expression levels but was also the most stable gene studied. In Figure 2, it can be observed that almost all genes presented average of log expression levels near 0 (the thick dashed line). Log difference >0 (as observed for *actin*) or <0 (as observed for *poly*) implies that variability in expression levels is significant, so the gene could be incorrectly used as reference gene for normalization. In this context, the *poly *gene presents the highest intertissue expression variation (SD = ± 0.65; see Figure 2). Thus, among the tested genes, *gapdh*, followed by *rpl7 *and *14-3-3*, showed the most stable expression over the investigated panel of five distinct coffee tissue/organ types.

The candidate genes were also ranked according to their M values using the geNorm program. The average expression stabilities (M values) of all tested genes were lower than 1.5, with *14-3-3 *and *actin *showing the most stable expression (data not shown). Although *actin *gene has shown highest stability following geNorm analysis, this gene presented the lowest expression profile according the BestKeeper analysis. Corroborating the previous analysis, *poly *remained the least stable gene.

As a whole, our analysis indicates that housekeeping genes are differently regulated in different tissues/organs of coffee plants and may exhibit variable expression patterns. The observed differences in gene expression ratios along a comprehensive panel of tissues/organs are consistent with the data presented by Barber et al. [1], Iskandar et al. [50] and Jain et al. [15]. Our results also provide evidences that normalizations to the expression level of a single gene in samples from distinct tissue types may induce to errors, thus corroborating previous studies [29,51,52].

### Comparison of the identified best reference genes to published data

In order to validate our potential candidate reference genes (*gapdh*, *rpl7 *and *14-3-3*), the expression stability of these genes under the influence of a specific biotic stress was investigated. In this case, the obtained results were compared to those dealing with similar coffee gene expression analysis but using *ubiquitin *as a reference gene for normalization [53-56].

The comparison was conduced by linear regressions analyzes of the CP difference (ΔCP) obtained from the assayed expression levels of the tested genes in leaves of *C. arabica *inoculated, or not (control plants), with *Hemileia vastatrix*. The average CP (*N *= 3) was calculated for each gene and the ΔCP (CP_inoculated leaf _- CP_non-inoculated leaf_) was determined for each time-point (8, 12 and 24 h after fungus inoculation).

**Figure 3:**
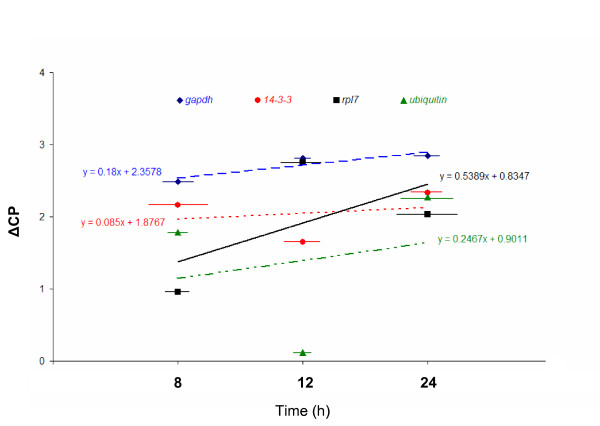
**Evaluation of the expression of selected reference genes during fungus infection**. The expression of selected reference genes (*gapdh*, *rpl7 *and *14-3-3*) and of a commonly used coffee normalization gene (*ubiquitin*) was monitored in leaves of *C. arabica *var. Mundo Novo inoculated with *Hemileia vastatrix*. The crossing point (CP) difference (ΔCP = CP_inoculated leaf _- CP_non-inoculated leaf_) was calculated for each time-point (8, 12 and 24 h after challenge by the rust fungus) to investigate the expression levels of each reference gene. The standard error of the triplicates for each time-point is indicated by horizontal bars.

As it can be observed in Figure 3, the regression lines for *14-3-3 *and *gapdh *have slopes close to zero (*14-3-3 *= 0.085; *gapdh *= 0.18), indicating similar expression levels in inoculated and non-inoculated leaves, and reinforcing their use as effective normalization genes [1,57,58]. In contrast, the slope value for *rpl7 *was significantly different from zero and higher than the one obtained for *ubiquitin *(*ubiquitin *= 0.2467; *rpl7 *= 0.5389), thus limiting its use as a normalization factor under biotic stress condition.

### Validation of data results in different developmental stages of flowers and coffee cherries

An additional validation step of the expression levels of *gapdh*, *14-3-3*, *rpl7 *and *ubiquitin *was performed using unpooled tissue samples from flower and cherry developmental series. The employed sample set is given in the Additional File 2.

**Figure 4:**
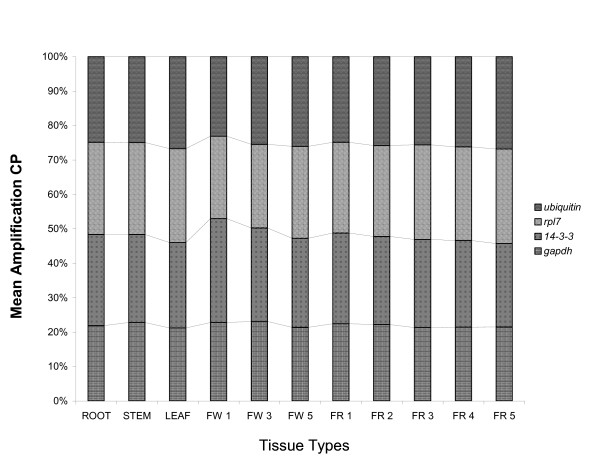
**Validation of the selected reference genes in samples from flowers and cherries at different developmental stages**. Comparison of gene contributions, by mean amplification crossing points (CP) represented in percentage, in each coffee tissue/organ type. The investigated tissue/organ sample set was: root, stem, leaf, three different stages of flower development (FW 1, FW 3 and FW 5) and five different kinds of coffee cherries (FR 1, FR 2, FR 3, FR 4 and FR 5).

In this assay, the highly expressed gene was *gapdh *($\bar{\text{CP}}$ = 21 cycles) followed by *ubiquitin *($\bar{\text{CP}}$ = 24 cycles), while *14-3-3 *and *rpl7 *presented the same mean CP (25 cycles). The comparison of gene contributions is present in Figure 4. As already observed, *gapdh *showed the greatest stability in expression among all coffee tissue/organ samples analyzed, while expression of *rpl7 *and *14-3-3 *varied the most, especially in flowers at stage 1 of development (see Figure 4 and Additional File 2). As mentioned earlier, *ubiquitin *was used as a standard reference gene for comparison.

### General remarks about the selected reference genes

According to these results, the gene encoding glyceraldeyde-3-phosphate dehydrogenase (GAPDH), an enzyme of glycolysis [59], outperformed all other reference genes tested and should therefore be considered a suitable reference gene for expression studies in arabica coffee plants. This observation corroborates the quantification of *gapdh *expression in different tissues of sugarcane [50] and *Eucalyptus *[20]. In contrast, several reports in human and animal systems have suggested that this reference gene has limitations for its use as internal control due to its marked variability of expression among tissue types [1,16,51,60].

The assayed *14-3-3 *gene also showed a stable expression (see Figure 2) that was supported by the descriptive analysis (see Table 1 and also Additional File 1) and confirmed in the biotic stress assay (see Figure 3). However, its expression presented some variation among different stages of coffee flower development (see Figure 4). Papini-Terzi et al. [19] recommended *14-3-3 *as a suitable reference gene for expression normalization in a wide range of tissue samples of sugarcane.

The gene encoding the transcription regulator and structural constituent of the 60S subunit of the cytosolic ribosome (*rpl7*) could also be used as an internal control in gene expression studies in *C. arabica*, due to its stability (see Figure 2) and acceptable variation among tissue/organ types (see Table 1). Our results are in agreement with previous published data for this gene since small variation among tissue types was detected by descriptive analysis (see Additional File 1). Nevertheless, in leaves of *C. arabica *inoculated with *H. vastatrix*, it was observed that the expression ratio of *rpl7 *(expressed by ΔCP) was not constant and the absolute value of *rpl7 *linear regression slope was superior to that observed for a commonly used coffee normalization gene (*ubiquitin*) (see Figure 3). In addition, this gene, like *14-3-3*, presented a variable expression level among different stages of coffee flower development (see Figure 4). In sugarcane, the relative expression of *rpl35-4*, a gene coding for the ribosomal L35-4 60S protein, was also reported to be stable [61]. These authors estimated the sugarcane leaf transcriptome using Serial Analysis of Gene Expression (SAGE) and reported that tag associated with the *rpl35-4 *transcript presented minimum variation among the analyzed SAGE libraries.

The remaining tested genes showed to be unsuitable as internal controls for normalization purposes in *C. arabica*.

## Conclusion

This study provides the most extensive collection of arabica coffee tissue/organ mRNA expression data for eight reference candidate genes. Our analysis evidenced stable levels of *gapdh*, *14-3-3 *and *rpl7 *mRNA in different *Coffea arabica *tissue/organ types. Consequently, these genes can be used for accurate and reliable normalization in future gene expression studies in coffee (e.g., they can be used as a reference for a target gene in a specific tissue or experimental condition). In this respect, we suggest *gapdh *as the most relevant reference gene for accurate normalization purposes in *C. arabica*, showing almost constant expression levels in the investigated experimental set-up.

Moreover, we have shown that depending on the reference inner control gene, the within-tissue variation of mRNA expression levels is generally small, whereas among tissues/organs the variation can be substantial. This indicates that normalizations to a single gene across different tissue types are unwise. Since the variation observed between normal tissues of different types may in part be due to the different metabolic demands of those tissues, comparisons within a tissue type between normal and diseased states are similarly unwise.

## Methods

### Plant material and growth conditions

Freshly harvested roots, stems, and leaves were obtained from ten 4 month-old coffee plants (*Coffea arabica *var. Mundo Novo IAC 388-17-1) grown under greenhouse conditions (28°C, 60% RH) in Campinas, São Paulo, Brazil. Flower and fruit samples, at different developmental stages, were collected from 4–5 year-old plants of var. Mundo Novo grown under field conditions at Botucatu and Campinas, São Paulo, Brazil (see Additional File 2). After harvesting, fresh tissue samples were frozen immediately in liquid nitrogen until RNA extraction.

### Biotic stress assay

For the biotic stress assay, equally-aged sets of *Coffea arabica *var. Mundo Novo plants were kept in growth chamber (16 h/8 h light/dark; 23°C; 70% RH) for at least one week, before being inoculated with the coffee leaf-rust fungus *Hemileia vastatrix *Berk and Br. race II, that elicits a compatible reaction in coffee. The urediniospores (100 mg) were harvested in a *C. arabica *field in Campinas, São Paulo, Brazil, and diluted in 10 ml of sterile water under dark conditions.

Leaves from the second pair of plagiotropic shoots from the apex of 4 month-old coffee plants were inoculated with an aqueous suspension of fresh urediniospores (10 mg/ml). To allow spore germination, the inoculated leaves were covered with a wet black plastic film for 24 h. Inoculated leaves were not detached from the plants. Mock-inoculated controls as well as non-inoculated controls were performed. The biological samples were obtained from three independent experiments.

Leaves were randomly sampled at different time-points after inoculation: 0, 8, 12, and 24 h, and immediately deep-frozen. To confirm the infection by the leaf-rust fungus, some inoculated leaves were maintained in plants.

### RNA isolation and quality controls

Tissue samples of 2.0 to 2.5 mg were weighed and ground to fine powder in liquid nitrogen using a pre-cooled mortar and pestle. Total RNA from the majority of the samples was extracted using TRIzol reagent (Invitrogen) according to manufacturer's instructions. Alternatively, total RNA from seeds was isolated by lithium chloride (LiCl) method, according to Mason and Schmidt [62]. Only RNA samples with 260/280 ratio between 1.9 and 2.1 and 260/230 ratio greater than 2.0 were used for subsequent analyses. The integrity of the RNA samples was also assessed on 1.0% agarose/formaldehyde gel electrophoresis.

### Reverse transcription

Five micrograms of total RNA were treated with DNAse I (Promega) and an aliquot of 500 ng of the treated RNA was reverse-transcribed using SuperScript First-Strand Synthesis System for RT-PCR (Invitrogen). Both were used following the manufacturer's instructions. The cDNA sample concentration was determined using the NanoDrop ND-1000 spectrophotometer (NanoDrop Technologies).

### Primer design

Primers were designed for *Coffea *orthologs of commonly used housekeeping genes representing distinct functional classes, identified by BLAST searches in the Brazilian Coffee EST database [63,64] as well as in the public coffee EST database (at the SOL site hosted by Cornell University [65]). For primer design, the Primer Express 2.0 software (PE Applied Biosystems, USA) with default parameters was employed. The accession numbers, gene description, primers sequences and amplicon lengths are shown in Table 2. All primer pairs produced a single product and amplified the target transcript with equal efficiency over a 1000-fold range of input material.

**Table 2:** Candidate reference genes and primer sequences used for quantitative PCR analysis

Gene name	Source gene ^a^	Gene description	Primer sequence ^b^	Amplicon length (bp)
*poly*	**SGN-U347154**	hexameric polyubiquitin	5' CGCTGACTACAATATCCAAAAGGA 3'	67
			5' CTGCATTCCACCCCTCAGA 3'	
*adh*	**SGN-U350348**	alcohol dehydrogenase class III	5' CCTCAAGCCGGCGAAGT 3'	55
			5' CTGTATGGCAGAGGGCAGTGT 3'	
*actin*	**SGN-U353034**	actin 7	5' AATTGTCCGTGACATCAAGGAA 3'	82
			5' TGAGCTGCTCTTGGCTGTTTC 3'	
*gapdh*	**SGN-U347734**	glyceraldehyde-3-phosphate dehydrogenase	5' TTGAAGGGCGGTGCAAA 3'	59
			5' AACATGGGTGCATCCTTGCT 3'	
*rpl7*	**SGN-U351477**	60S ribosomal protein L7	5' CATTCGAGGTATCAATGCTATGCA 3'	66
			5' TGTCTCAGGCGCAGAAGCT 3'	
*ccs*	**SGN-U350284**	caffeine synthase 1	5' CAATGCCCGGCTCTTTCTAC 3'	68
			5' GTAACAAGAGTGTAAAAAATGCATGGA 3'	
*cys*	**SGN-U352616**	cysteine proteinase	5' GCGATCGCTACCGTCCAA 3'	63
			5' CTTTTTCTCTCCAGTCAATGGAGTT 3'	
*14-3-3*	**SGN-U356404**	14-3-3 protein	5' TGTGCTCTTTAGCTTCCAAACG 3'	75
			5' CTTCACGAGACATATTGTCTTACTCAAA 3'	

^a ^Unigene accession number according to the SOL Genomics Network [65]

^b ^Forward (upper line) and reverse (lower line) primer sequences

### Quantitative PCR

The PCR mixture contained 5 μl of a 1:10 dilution of the synthesized cDNA, primers to a final concentration of 700 nM each, 17.5 μl of the SYBR Green PCR Master Mix (Applied Biosystems, USA) and PCR-grade water up to a total volume of 35 μl. The mixes were homogenized and split in three samples of 10 μl, thus each gene reaction was performed in triplicate. PCR reactions in the absence of template were also performed as negative controls for each primer pair. An equimolar pool of cDNA samples of five coffee tissue/organ types (root, stem, leaf, flower, and fruit) was prepared to be used as a common reference for all qPCR. The quantitative PCRs were performed employing the ABI Prism 7300 Sequence Detection System (PE Applied Biosystems, USA). All PCR reactions were performed under the following conditions: 2 min at 50°C, 10 min at 95°C, and 45 cycles of 15 s at 95°C and 1 min at 65°C in 96-well optical reaction plates (Applied Biosystems, USA). Confirmation of amplicon specificity was based on the dissociation curve at the end of each run and by product visualization after electrophoresis on an 8% polyacrylamide gel.

### Analysis of candidate reference genes

To estimate the expression variation level of the eight candidate genes (*actin*, *adh*, *ccs*, *14-3-3*, *gapdh*, *poly*, *rpl7*, or *cys*) over a five coffee tissue/organ sample set (root, stem, leaf, flower, or fruit), the BestKeeper descriptive statistical method [45,66] was applied.

To access the levels of gene expression for each gene in the different coffee tissue types, the method described by Ramakers et al. [67] with modifications was used. Optic data were exported from 7300 Real-Time PCR System (PE Applied Biosystems, USA) into MS Excel. Four cycles at the exponential phase, near and including the crossing point (CP), were used. The fluorescence data were logarithmically transformed, and pasted into statistical software package (SAS version 8e, SAS Institute, Cary, NC, USA) for linear regression analysis, including determination of intercepts, slopes (x), PCR efficiency (*E *= 10^slope^) and their respective standard errors and correlation coefficients (R^2^). The gene expression values (x-fold) were obtained according to a mathematical model proposed by Pfaffl [68]: x-fold = *E*_reference gene _^ΔCP^, where ΔCP = CP_pool of tissues _- CP_tissue sample_.

Knowing the expression levels, the stability value was assessed utilizing NormFinder [8,49] and geNorm [17,69] programs.

## Abbreviations

qPCR: Quantitative PCR; *poly*: hexameric polyubiquitin; *adh*: alcohol dehydrogenase class III; *actin*: actin 7; *rpl7*: 60S ribosomal protein L7; *ccs*: caffeine synthase 1; *gapdh*: glyceraldehyde-3-phosphate dehydrogenase; *cys*: cysteine proteinase; CP: crossing point.

## Authors' contributions

CFB-C and IGM conceived and designed this study. FES performed the biological assays. CFB-C and FES carried out the molecular genetic studies, participated in the qPCR experiments (acquisition, analysis and interpretation of data). MPM and IGM contributed with reagents/materials/analysis tools. IGM coordinated the study. CFB-C wrote the manuscript. All authors contributed, read, corrected and approved the final manuscript. The authors declare no conflict of interest in this work.

## Supplementary Material

Additional file 1 **Results from Bestkeeper descriptive statistical analysis**. The data provided represent the descriptive statistics, based on crossing point (CP) values, for the expression analyses of the candidate reference genes in the five distinct coffee plant tissue/organ set. Click here for file

Additional file 2 **Coffee tissue/organ sample set (*****Coffea arabica *****var. Mundo Novo – IAC 388-17-1) used in the present study**. Freshly harvested roots, stems, and leaves were obtained from 4 month-old coffee plants grown under greenhouse conditions (28°C, 60% RH) in Campinas, São Paulo, Brazil. Flower and fruit samples, at different developmental stages, were collected from 4–5 year-old field grown plants in Botucatu and in Campinas, São Paulo, Brazil. After harvesting, fresh tissue samples were frozen immediately in liquid nitrogen until RNA extraction. Click here for file
