# Supplementary material for: Identification of suitable internal control genes for expression studies in Coffea arabica under different experimental conditions
Source: BMC Mol Biol. 2009 Jan 6;10:1. doi: 10.1186/1471-2199-10-1 (PMC2629470; doi:10.1186/1471-2199-10-1)
Supplement: Additional file 1 — Results from Bestkeeper descriptive statistical analysis. The data provided represent the descriptive statistics, based on crossing point (CP) values, for the expression analyses of the candidate reference genes in the five distinct coffee plant tissue/organ set. [file 1471-2199-10-1-S1.doc]

| Gene | Factor | Pool | Root | Stem | Leaf | Flower | Fruit |
| --- | --- | --- | --- | --- | --- | --- | --- |
| *actin* | N | 3 | 3 | 3 | 3 | 3 | 3 |
|  | GM [CP] | 32,41 | 31,40 | 32,98 | 33,34 | 34,35 | 31,63 |
|  | AM [CP] | 32,41 | 31,40 | 32,98 | 33,34 | 34,35 | 31,63 |
|  | Min [CP] | 32,17 | 31,26 | 32,78 | 33,28 | 34,15 | 31,51 |
|  | Max [CP] | 32,72 | 31,54 | 33,17 | 33,45 | 34,76 | 31,81 |
|  | SD [±CP] | 0,28 | 0,20 | 0,20 | 0,10 | 0,35 | 0,16 |
|  | CV [%CP] | 0,87 | 0,62 | 0,59 | 0,29 | 1,02 | 0,52 |
|  |  |  |  |  |  |  |  |
|  | Min (x-fold) |  | 1,85 | 2,71 | 1,83 | 0,80 | 2,39 |
|  | Max (x-fold) |  | 1,85 | 3,11 | 2,64 | 1,38 | 3,87 |
|  | SD (±x-fold) |  | 0.00 | 0.22 | 0.41 | 0.32 | 0.78 |
| *adh* | N | 3 | 30 | 3 | 3 | 3 | 3 |
|  | GM [CP] | 29.55 | 28.64 | 30.11 | 29.78 | 31.12 | 28.89 |
|  | AM [CP] | 29.55 | 28.64 | 30.12 | 29.78 | 31.12 | 28.89 |
|  | Min [CP] | 28.91 | 28.45 | 30.02 | 29.50 | 31.02 | 28.42 |
|  | Max [CP] | 29.88 | 28.99 | 30.21 | 30.06 | 31.25 | 29.43 |
|  | SD [±CP] | 0.56 | 0.30 | 0.13 | 0.40 | 0.12 | 0.51 |
|  | CV [%CP] | 1.89 | 1.05 | 0.45 | 1.33 | 0.38 | 1.76 |
|  |  |  |  |  |  |  |  |
|  | Min (x-fold) |  | 0.72 | 0.81 | 0.97 | 0.70 | 0.80 |
|  | Max (x-fold) |  | 1.43 | 1.65 | 1.27 | 1.65 | 1.13 |
|  | SD (±x-fold) |  | 0.36 | 0.60 | 0.21 | 0.52 | 0.19 |
| *14-3-3* | N | 3 | 3 | 3 | 3 | 3 | 3 |
|  | GM [CP] | 29.38 | 29.68 | 29.24 | 28.94 | 31.21 | 29.14 |
|  | AM [CP] | 29.38 | 29.68 | 29.24 | 28.94 | 31.21 | 29.14 |
|  | Min [CP] | 29.28 | 29.30 | 29.18 | 28.81 | 31.01 | 29.07 |
|  | Max [CP] | 29.52 | 29.92 | 29.31 | 29.04 | 31.54 | 29.20 |
|  | SD [±CP] | 0.12 | 0.34 | 0.07 | 0.12 | 0.29 | 0.07 |
|  | CV [%CP] | 0.42 | 1.13 | 0.22 | 0.41 | 0.92 | 0.23 |
|  |  |  |  |  |  |  |  |
|  | Min (x-fold) |  | 0.84 | 0.96 | 0.98 | 0.94 | 0.96 |
|  | Max (x-fold) |  | 1.13 | 1.03 | 1.04 | 1.13 | 1.03 |
|  | SD (±x-fold) |  | 0.16 | 0.03 | 0.03 | 0.11 | 0.04 |
| *ccs* | N | 3 | 3 | 3 | 3 | 3 | 3 |
|  | GM [CP] | 26.35 | 27.37 | 27.01 | 26.80 | 26.23 | 25.90 |
|  | AM [CP] | 26.35 | 27.37 | 27.02 | 26.80 | 26.26 | 25.92 |
|  | Min [CP] | 26.12 | 27.08 | 26.95 | 26.63 | 25.11 | 25.17 |
|  | Max [CP] | 26.47 | 27.66 | 27.08 | 27.04 | 27.40 | 27.40 |
|  | SD [±CP] | 0.20 | 0.41 | 0.09 | 0.22 | 1.62 | 1.28 |
|  | CV [%CP] | 0.77 | 1.50 | 0.34 | 0.80 | 6.17 | 4.96 |
|  |  |  |  |  |  |  |  |
|  | Min (x-fold) |  | 0.95 | 0.95 | 0.83 | 0.44 | 0.56 |
|  | Max (x-fold) |  | 1.18 | 1.17 | 1.10 | 2.12 | 2.87 |
|  | SD (±x-fold) |  | 0.16 | 0.15 | 0.14 | 1.19 | 1.29 |
| *gapdh* | N | 3 | 3 | 3 | 3 | 3 | 3 |
|  | GM [CP] | 22.56 | 22.05 | 23.67 | 23.74 | 23.08 | 21.73 |
|  | AM [CP] | 22.56 | 22.06 | 23.68 | 23.75 | 23.08 | 21.73 |
|  | Min [CP] | 22.47 | 22.05 | 23.49 | 23.72 | 23.05 | 21.66 |
|  | Max [CP] | 22.67 | 22.06 | 23.86 | 23.77 | 23.11 | 21.80 |
|  | SD [±CP] | 0.10 | 0.01 | 0.26 | 0.04 | 0.03 | 0.10 |
|  | CV [%CP] | 0.45 | 0.03 | 1.11 | 0.15 | 0.13 | 0.46 |
|  |  |  |  |  |  |  |  |
|  | Min (x-fold) |  | 1.01 | 0.96 | 1.03 | 0.96 | 1.01 |
|  | Max (x-fold) |  | 1.05 | 1.08 | 1.05 | 1.03 | 1.06 |
|  | SD (±x-fold) |  | 0.02 | 0.09 | 0.02 | 0.04 | 0.03 |
| *poly* | N | 3 | 3 | 3 | 3 | 3 | 3 |
|  | GM [CP] | 27.74 | 26.79 | 30.07 | 29.85 | 28.82 | 27.08 |
|  | AM [CP] | 27.75 | 26.79 | 30.07 | 29.86 | 28.83 | 27.08 |
|  | Min [CP] | 27.45 | 26.51 | 29.93 | 29.68 | 28.50 | 27.01 |
|  | Max [CP] | 28.22 | 27.07 | 30.21 | 30.03 | 29.15 | 27.15 |
|  | SD [±CP] | 0.41 | 0.40 | 0.20 | 0.25 | 0.46 | 0.10 |
|  | CV [%CP] | 1.49 | 1.48 | 0.66 | 0.83 | 1.59 | 0.37 |
|  |  |  |  |  |  |  |  |
|  | Min (x-fold) |  | 1.01 | 1.21 | 1.16 | 0.97 | 1.17 |
|  | Max (x-fold) |  | 1.51 | 1.46 | 1.57 | 1.74 | 1.18 |
|  | SD (±x-fold) |  | 0.35 | 0.18 | 0.29 | 0.54 | 0.01 |
| *rpl7* | N | 3 | 3 | 3 | 3 | 3 | 3 |
|  | GM [CP] | 30.58 | 30.80 | 31.93 | 30.71 | 30.38 | 29.96 |
|  | AM [CP] | 30.58 | 30.81 | 31.93 | 30.71 | 30.39 | 29.96 |
|  | Min [CP] | 30.38 | 30.73 | 31.90 | 30.47 | 30.37 | 29.73 |
|  | Max [CP] | 30.78 | 30.88 | 31.96 | 31.08 | 30.40 | 30.19 |
|  | SD [±CP] | 0.20 | 0.11 | 0.04 | 0.33 | 0.02 | 0.33 |
|  | CV [%CP] | 0.65 | 0.34 | 0.13 | 1.06 | 0.07 | 1.09 |
|  |  |  |  |  |  |  |  |
|  | Min (x-fold) |  | 1.05 | 1.02 | 0.93 | 1.01 | 0.98 |
|  | Max (x-fold) |  | 1.08 | 1.11 | 1.11 | 1.12 | 1.15 |
|  | SD (±x-fold) |  | 0.02 | 0.06 | 0.10 | 0.08 | 0.12 |
| *cys* | N | 3 | 3 | 3 | 3 | 3 | 3 |
|  | GM [CP] | 26.05 | 25.24 | 28.43 | 27.53 | 26.55 | 25.72 |
|  | AM [CP] | 26.06 | 25.24 | 28.44 | 27.54 | 26.55 | 25.72 |
|  | Min [CP] | 26.04 | 25.18 | 28.32 | 27.50 | 26.36 | 25.58 |
|  | Max [CP] | 26.07 | 25.32 | 28.55 | 27.57 | 26.82 | 25.80 |
|  | SD [±CP] | 0.02 | 0.07 | 0.16 | 0.05 | 0.24 | 0.12 |
|  | CV [%CP] | 0.08 | 0.29 | 0.57 | 0.18 | 0.91 | 0.48 |
|  |  |  |  |  |  |  |  |
|  | Min (x-fold) |  | 0.97 | 0.94 | 0.99 | 0.90 | 0.92 |
|  | Max (x-fold) |  | 0.98 | 1.05 | 1.01 | 0.94 | 1.04 |
|  | SD (±x-fold) |  | 0.00 | 0.08 | 0.02 | 0.03 | 0.08 |

Abbreviations: N: number of samples; CP: crossing-point; GM [CP]: geometric CP mean; AM [CP]: arithmetic CP mean; Min [CP] and Max [CP]: CP threshold values; SD [± CP]: CP standard deviation; CV [%CP]: variance coefficient expressed as percentage of CP level; Min [x-fold] and Max [x-fold]: threshold expression levels expressed as absolute x-fold over- or under-regulation coefficient; SD [± x-fold]: standard deviation of absolute regulation coefficient.
