# Supplementary material for: Identification of suitable internal control genes for expression studies in Coffea arabica under different experimental conditions
Source: BMC Mol Biol. 2009 Jan 6;10:1. doi: 10.1186/1471-2199-10-1 (PMC2629470; doi:10.1186/1471-2199-10-1)
Supplement: Additional file 2 — Coffee tissue/organ sample set (Coffea arabica var. Mundo Novo – IAC 388-17-1) used in the present study. Freshly harvested roots, stems, and leaves were obtained from 4 month-old coffee plants grown under greenhouse conditions (28°C, 60% RH) in Campinas, São Paulo, Brazil. Flower and fruit samples, at different developmental stages, were collected from 4–5 year-old field grown plants in Botucatu and in Campinas, São Paulo, Brazil. After harvesting, fresh tissue samples were frozen immediately in liquid nitrogen until RNA extraction. [file 1471-2199-10-1-S2.doc]

A B C

D E F G H I

J K L M N

The tissues analyzed are represented by: A= root, B= stem, C= leaf, D and E= first stage of flower development (tight buds; no swelling; vegetative buds indistinguishable from floral buds), F= inflorescence representing the second and third stages of floral bud development, G= closed floral bud corresponding to stage 4 of flower development, H= semi-open flowers corresponding to stage 5 of flower development, I= stage 6 of flower development (mature flowers), J and K= maternal phase of fruit development (perisperm), L= endosperm development, M= pericarp maturation and storage phase, N= mature cherry. D to I = classification according to Majerowicz and Söndahl [70], Pezzopane et al. [71] and Morais et al. [72], J to N= classification according to Morais et al. [72] and de Castro et al. [73].
